# Identifying Hybrid DDoS Attacks in Deterministic Machine-to-Machine Networks on a Per-Deterministic-Flow Basis

**DOI:** 10.3390/mi12091019

**Published:** 2021-08-26

**Authors:** Yen-Hung Chen, Yuan-Cheng Lai, Kai-Zhong Zhou

**Affiliations:** 1 Department of Information Management, National Taipei University of Nursing and Health Sciences, Taipei 112, Taiwan; 2 Department of Information Management, National Taiwan University of Science and Technology, Taipei 106, Taiwan; laiyc@cs.ntust.edu.tw (Y.-C.L.); kaizhongchou@gmail.com (K.-Z.Z.)

**Keywords:** DetNet, SDN, Hybrid DDoS attacks, flow-based detection

## Abstract

The Deterministic Network (DetNet) is becoming a major feature for 5G and 6G networks to cope with the issue that conventional IT infrastructure cannot efficiently handle latency-sensitive data. The DetNet applies flow virtualization to satisfy time-critical flow requirements, but inevitably, DetNet flows and conventional flows interact/interfere with each other when sharing the same physical resources. This subsequently raises the hybrid DDoS security issue that high malicious traffic not only attacks the DetNet centralized controller itself but also attacks the links that DetNet flows pass through. Previous research focused on either the DDoS type of the centralized controller side or the link side. As DDoS attack techniques are evolving, Hybrid DDoS attacks can attack multiple targets (controllers or links) simultaneously, which are difficultly detected by previous DDoS detection methodologies. This study, therefore, proposes a Flow Differentiation Detector (FDD), a novel approach to detect Hybrid DDoS attacks. The FDD first applies a fuzzy-based mechanism, Target Link Selection, to determine the most valuable links for the DDoS link/server attacker and then statistically evaluates the traffic pattern flowing through these links. Furthermore, the contribution of this study is to deploy the FDD in the SDN controller OpenDayLight to implement a Hybrid DDoS attack detection system. The experimental results show that the FDD has superior detection accuracy (above 90%) than traditional methods under the situation of different ratios of Hybrid DDoS attacks and different types and scales of topology.

## 1. Introduction

As the 5th Generation (5G) of mobile communication promoted telecommunication technologies across different domains and achieved initial success, the 3rd-Generation Partnership Project (3GPP) began investigating advanced features to pave the way for 6G [1]. One of these features is the Deterministic Network (DetNet) to cope with the issue that conventional IT infrastructure cannot efficiently handle latency-sensitive data [1,2,3,4]. The DetNet transforms time-critical flows into Deterministic Flows (DetNet Flows) and enables guaranteed bandwidth, latency, and corresponding features, which are germane to transport time-sensitive data. By doing so, the DetNet can migrate time-critical applications from application-specific proprietary networks to packet technologies in the conventional IP-based infrastructure [4]. Due to the potentiality of the DetNet in 6G, numerous DetNet industrial automation use cases have been identified, including machine-to-machine communication in building automation systems and in the mining industry, and several corresponding methodologies have also been proposed [3]. However, several security issues have subsequently arisen [3], such as delay attacks, spoofing, reconnaissance, and, of course, Hybrid Distributed Denial of Service (Hybrid DDoS). In this study, we focus on the issue of Hybrid DDoS.

Hybrid DDoS means high malicious traffic not only attacks the DetNet centralized controller itself but also attacks the links that DetNet flows pass through. On the one hand, Hybrid DDoS follows the conventional DDoS attacking pattern that exhausts controller resources by using a high volume of legitimate traffic, causing the controller operation to be significantly delayed or interrupted. This type of DDoS attack is also called a Server-Exhausted Attack (SEA) [5,6,7,8,9,10,11,12,13,14,15,16]. On the other hand, Hybrid DDoS also adopts a new type of DDoS attack, the Link Flooding Attack (LFA) [17,18]. The LFA congests key links, called target links, to isolate servers in the target area where the cloud machines are located from clients. Hybrid DDoS attacks can easily sneak into a network under the monitoring of the current DDoS detection system. For example, the attacker can paralyze the DetNet by only congesting 60% capacity of the targeted links and 50% capacity of the centralized DetNet controller without being detected because both attacking behaviors may be treated as normal traffic patterns.

Numerous studies have explored how to detect a single type of DDoS attack, i.e., an SEA [8,9,10,11,12,13,14,15,16] or LFA [19,20,21,22,23,24]. However, the presence, magnitude, and synergy effect of hybrid attacks that launch an SEA and LFA at the same time have not been investigated. Hybrid attacks, on the one hand, apply the LFA to congest 60% capacity of target links, and on the other hand, allow the SEA to traverse noncongested target links to consume 40% capacity of the servers in the target area, causing a synergy effect where the service is interrupted. The synergy effect is an interaction or cooperation between the SEA and LFA, giving rise to a whole that is greater than the simple sum of its parts on the availability of the network. In this situation, the conventional LFA detector observes that 40% capacity of target links is available and may ignore the LFA event. Furthermore, the SEA detector calculates the server has 40% capacity to satisfy the request and may disregard SEA traffic. Under hybrid attacks, the reason causing the out-of-service effect is clearly not attributed to the SEA or the LFA. Therefore, current detection systems that can detect an SEA or LFA are not applicable to detect hybrid attacks and the corresponding synergy effects. A new detection approach that can detect Hybrid DDoS attacks and the corresponding synergy effects should be studied.

This study, therefore, proposes a Flow Differentiation Detector (FDD) to detect Hybrid DDoS attacks in the DetNet. The FDD adopts a two-step detection scheme: (1) it first applies a fuzzy-based feature extraction methodology to determine the most valuable links for the DDoS link/server attacker, and then (2) it applies a heuristic approach to detect the network behaviors generated from the SEA and LFA. The FDD observes two common characteristics of the SEA and LFA: (1) they must traverse through the target links, and (2) their source IP address or destination IP address must be the servers located in the target area. When hybrid attacks occur, the number of DetNet flows that pass through the servers in the target area increases, while the number of flows that depart from the servers in the target area remains almost fixed. The FDD is also implemented in OpenDayLight, which is a typical Software-Defined Networking (SDN) controller to observe the performance of the FDD in detecting hybrid attacks in an SDN environment.

The contributions of the FDD are threefold: (1) to implement a hybrid attack in OpenDayLight, which is a typical Software-Defined Networking (SDN) controller, to observe and ensure the existence of hybrid attacks and synergy in an SDN environment; (2) to provide a conceptual solution to detect hybrid attacks in the DetNet; and (3) to evaluate the performance of current solutions and discuss a possible development path to defend against hybrid attacks.

This study is organized as follows. Section 2 reviews related work, including SEA detection and LFA detection. The system model and proposed method, the FDD, are described in Section 3 and Section 4, respectively. The experimental environment and results are discussed in Section 5. The conclusions are given in Section 6.

## 2. Background

This section describes the various types of DDoS attacks, including SEAs, LFAs, and hybrid attacks, and the corresponding detection methods.

### 2.1. Server-Exhausted Attacks and Detection Methods

SEAs exploit server vulnerability and generate a large number of requests to quickly exhaust the targeted server’s resources. Therefore, a server lacking resources cannot respond to legitimate requests and may even crash. A typical SEA includes SYN flooding attacks [8,10,11], DNS flooding attacks [9,12,13], and HTTP flooding attacks [14,15,16].

SYN flooding attacks that transmit TCP connections and ACK packets are not returned, causing the server to store all these requests and, eventually, run out of resources. Therefore, [8] calculates the number of SYN packets collected by a server and analyzes the response rate of an ACK packet, thereby representing the level of internal server resource damage. Reference [10] calculates the entropy between the source IP and destination IP of a TCP packet and analyzes the network usage behavior. Entropy represents the degree of concentration of information under a SEA, and a large number of centralized nodes request the target server. Reference [11] uses Network Function Virtualization (NFV) to design a dynamic resource allocation mechanism to detect attacks. 

DNS flooding attacks send a large number of nonexistent domain-name requests to the DNS, causing DNS server failure. To detect DNS flooding, [9] calculates the repetition rate of the source IP and the destination IP of flows through a DNS. Reference [12] uses Supervised Machine Learning (SML) to analyze all requests for accessing a DNS and determines those sending a large number of IP locations for nonexistent domain names to block these requests. Reference [13] uses machine learning to implement a system to detect abnormal packets and automatically block attack packets.

An HTTP flooding attack means that the clients interact with the server and send HTTP requests to compel the server to allocate as many resources as possible to serve the attack, thus denying legitimate users access to the server’s resources. Reference [14] uses server logs to train which IP is abnormal. Reference [15] uses the correlation between IP address and traffic for detection in a Named Data Network (NDN). Reference [16] builds a model using anomaly-based detection approaches by tracing browsing logs to detect attacks.

### 2.2. Link Flooding Attacks and Detection Methods

LFAs exploit vulnerabilities in topology by generating legitimate traffic nearby the target area to congest key links. If all target links are congested, the target area cannot receive requests from an external network, and the service in the target area is therefore unreachable. LFAs comprise two kinds of attacks, i.e., Coremelt attacks and Crossfire attacks, where the former represents having a single target link, while the latter represents having multiple target links. For example, Reference [19] uses end-to-end and hop-by-hop network measurement techniques; Reference [20] tests node-to-node traffic variation to capture abnormal links through bandwidth usage, delay time, and packet loss rate to detect an attack in traditional networks; Reference [21] calculates traffic density to find target links and then monitors these target links’ utilization; References [22,23] builds a centralized network architecture and periodically observes the target area network traceroute packet to eliminate malicious traffic before an attack occurs.

### 2.3. DetNet and the Hybrid Attacks and Detection Methods

The DetNet applies flow virtualization to satisfy time-critical flow requirements. The DetNet virtualizes heterogeneous data flows in order to (1) preserve the properties of these flows over a converged IP-based infrastructure and (2) be backward compatible with statistically multiplexed IP-based traffic. Therefore, two tasks of DetNet flow virtualization are designed. First, the DetNet duplicates and eliminates packets over noncongruent routing paths to achieve a sufficiently high delivery ratio and satisfy the application’s QoS requirement [2]. Second, the DetNet applies central controllers, i.e., Software-Defined Network (SDN) controllers, to enable a fully scheduled operation orchestrated to integrate virtualized DetNet flows in conventional IP-based implementations. The DetNet controller frequently obtains flow tables from switches with the OpenFlow protocol or measures network topology with the Link Layer Discovery Protocol (LLDP) and the Broadcast Domain Discovery Protocol (BDDP) in the control plane. These two tasks inevitably incur that DetNet flows and conventional flows share the same physical resources and interact/interfere with each other [3]. Several security issues subsequently arise [3], such as delay attacks, spoofing, reconnaissance, and, of course, Hybrid Distributed Denial of Service (Hybrid DDoS). This study focuses on the issue of Hybrid DDoS.

Most studies focus on detecting a single type of DDoS attack, i.e., SEA or LFA, in a traditional network rather than DetNet. Reference [24] explores hybrid attacks in SDN, which launch an SEA and LFA interrelatedly. It uses correlation to cluster traffic to determine whether it is an SEA and measures the target link utilization to determine whether it is an LFA. The study also integrates two types of attacks in one system but ignores hybrid attacks that can launch an SEA and LFA at the same time. The Hybrid DDoS not only attacks the DetNet centralized controller itself but also attacks the links that DetNet flows pass through. Therefore, a new detection approach that can detect Hybrid DDoS attacks should be studied.

The comparison of the studies mentioned above is summarized in Table 1. Type refers to the type of DDoS. Environment refers to which network environment the detection executes. Attack refers to where the attacks are detected. Method refers to the paper solution concept. Flow/packet-based refers to the solution depending on the features. Flow-based refers to packet transmission route traces such as IPFIX [25], and packet-based refers to packet payload. Dataset refers to the paper test using a real or simulated dataset.

## 3. DetNet Environment Setting and Notation Description

**Figure 1:**
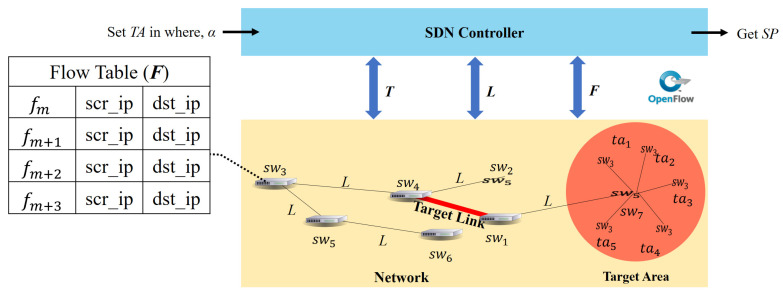
System model.

The DetNet is a centralized network architecture for managing network node behavior and topology information. Figure 1 shows the system model of this study. This study applies an SDN controller as a supervisor to manage the DetNet environment and control all switches that store all flow tables. When Hybrid DDoS attackers launch attacks, the behavior of flows is recorded in the flow table. The controller can obtain the links (*L*) and switches of all flow tables in the network.

Table 2 shows the notations used in this study. *T* represents the size of the topology, *SW* represents the switches, *TA* represents the servers in the target area, *F* represents all flows ($f_{m}^{}$) in the flow table in the SDN switches, α represents how to set the links as target links, *L* represents links in SDN, *TL* represents target links in SDN, and SP represents the system that determines the attack standard.

## 4. Proposed Method Flow Differentiation Detector

This study attempts to maximize detection accuracy under hybrid attacks in the DetNet. The basic assumption is that the target link can be the observation point to detect both an SEA and LFA because it can capture the most flows through the target area. Regardless of which flows accessing or responding are for the target area, these flows will pass through the target links with a high probability. In order to detect the network behaviors generated from the SEA and LFA, the hybrid attack detector is suggested to adopt their common features, namely the number of target flows, which have two characteristics: (1) they must traverse through the target links, and (2) their source IP address or destination IP address must be the servers located in the target area. Furthermore, when hybrid attacks occurred, we observed a phenomenon where the number of flows that passed through the servers in the target area increased, while the number of flows that departed from the servers in the target area was almost fixed. However, under normal traffic, the number of flows that departed from the servers increased when the number of flows that passed through the servers increased because the servers can provide normal services.

As discussed above, this study proposes a Flow Differentiation Detector (FDD). The FDD adopts a two-step detection scheme to detect Hybrid DDoS attacks in the DetNet. First, the FDD uses a fuzzy-based feature extraction methodology to determine the most valuable links and locate the targeted area of the Hybrid DDoS attacker. Second, the FDD statistically evaluates and detects the network behaviors generated from the SEA and LFA based on the observation of the target links.

### 4.1. Step 1: Locate the Target Links

For the first step, to locate the targeted links, the FDD calculates the link weight of each link according to Formula (1) and selects the top 20% links as the target links based on the Pareto principle, where roughly 80% of consequences come from 20% of causes [21]. Link weight (*W*) represents the importance of the links enclosed in an area, $\sum_{m=1}^{M}f_{m.dst}$ is the number of flows in the destination IP equal to the target area $,\;\sum_{m=1}^{M}f_{m.scr}$ is and the number of flows in the source IP equal to the target area. We multiplied the two values to obtain the link weight and sorted the link from high to low by link weight.

(1) $$\mathrm{Link}\;\mathrm{Weight}\left(W\right)=\overset{M}{\underset{m=1}{\sum }}f_{m.dst}\times \overset{M}{\underset{m=1}{\sum }}f_{m.scr}$$

After finding the target link, we selected the switch that was farthest from the target area at the end to the ends of the target link. It was necessary to use the end of the target link to measure the service performance considered attacked by an SEA and LFA. We merged the flow tables from these switches and divided these flows in the target link into input flows and output flows. The input flows with a destination IP equal to the target area represent an external request to access the target area, and the output flows with a source IP equal to the target area represent a service response from the target area. Regardless of the type of attack, the number of input flows will increase, and the number of output flows will maintain a value or increase slowly. Therefore, we calculated the frequency of the differentiation between the number of input flows and output flows.

The OpenFlow protocol supports IP Flow Information Export (IPFIX) [25]. We used common features called flow keys, such as five tuples, source and destination IP addresses, source and destination port number, and communication protocols (ip_src, ip_dst, port_src, port_dst, proto). We monitored the behavior of the network by flow traffic transmission and defined that flows with the same source IP addresses, destination IP addresses, and protocols will be considered the same flow.

**Figure 2:**
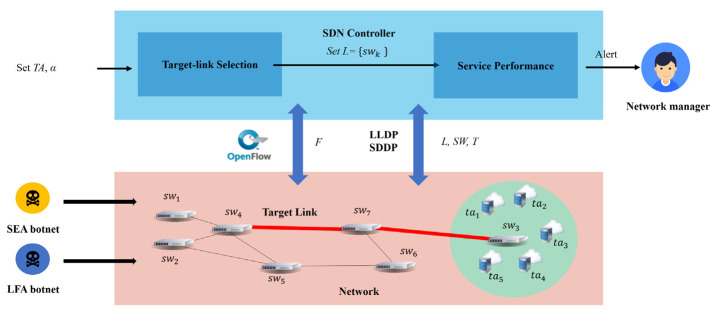
FDD process schematic diagram.

In order to detect hybrid attacks, it was necessary to calculate the flow’s traffic attacking the target area. For this purpose, we used an SDN controller to complete the process and calculated the flow differentiation by the number of flows to be the service performance in the network [10,12,14,15] and the topology to find target links and set the observation points. Figure 2 shows the FDD process schematic diagram.

### 4.2. Step 2: Statistically Detect Hybrid DDoS Attacks Observed from the Target Links

The FDD statistically evaluates and detects the network behaviors generated from the SEA and LFA based on the observation of the target links in the second step. The FDD applies a chi-squared test, which is a statistical hypothesis test to determine whether there is a statistically significant difference between the influx of expected flows and the influx of observed flows. Influx refers to the difference between the traffic flow in the targeted area and the traffic flow outside the targeted area. If the testing result is significantly different, it can be determined that the target area is attacked, and an alert is sent to the network manager. The formula (2) for the chi-square statistic $x_{c}^{2}$ is:

(2) $$x_{c}^{2}=\sum \frac{{\left(O_{i}-E_{i}\right)}^{2}}{E_{i}}$$

where *c* is the degrees of freedom, and *O_i_* is the evaluated sample calculated by formula (3)

(3) $$O_{i}=\left(SP_{input}\times k-SP_{output}\right)$$

*E_i_* is the $O_{i}\;$ observed beforehand, *i* is the *i*-th data in the sampled time period, and *k* is used to moderate the sensitivity of testing. The *k* value is set to 1.0 in this study and can be modified by the network administrator based on their security policy. The chi-square test $x_{c}^{2}$ is a *p*-value that evaluates whether the test result is significantly a hybrid attack or not.

**Figure 3:**
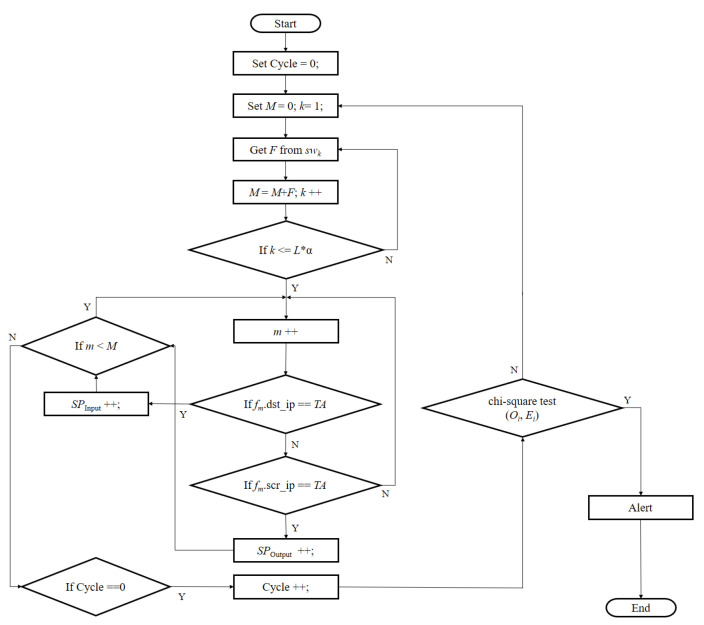
Flowchart of FDD methodology.

The detailed procedure, as shown in Figure 3, first considers the first time to detect a situation, so we define the variable (Cycle) to identify the number of detections; then, the FDD selects above these switches from the target links’ end. From these switches ($SW_{k}$), the flow tables (F) merge the flow tables into one table *M*. Third, we avoid causing measurement error, selecting existing flows. Other flows in the contrast source IP and the destination IP are measuring flows, and the remaining flows are deleted. Fourth, the large table (M) classifies the number of input (*SP_input_*) and output (*SP_output_*) flows. The last step applies a chi-squared test to determine whether there is a statistically significant difference between the observed $O_{i}$ and the expected $E_{i}$. If the testing result is significantly different, it can be determined that the target area is attacked, and an alert is sent to the network manager.

## 5. Implementation

The experiment of this study establishes a hybrid attack detection system based on the FDD in SDN. It is implemented in Java, deployed on OpenDayLight, which is a popular open-source SDN controller, and obtains flow information through OpenDayLight’s REST APIs so that it will not introduce additional loading overhead to the controller. There are two main modules in this system: (1) the Target Link Selection Module (TSM) and (2) the Service Performance Module (SPM).

The TSM is responsible for calculating which links will be selected as target links. It also calculates the link weight using Formula (1) in [21]. The TSM obtains all flows through the target area in the network to sort those links by link weight from high to low weight. On the other hand, it also needs to know the switch and link positions in the topology and consider the topology and link weight together to the target links. The primary REST APIs used by the TSM mainly include APIs1 and APIs2 to receive all switch flow tables, where APIs1 and APIs2 are as follows:

- APIs1: /restconf/operational/network-topology:network-topology;
- APIs2: /restconf/operational/opendaylight-inventory:nodes/node/switch/table.

The SPM applies a chi-square test to evaluate the differentiation between input and output flows to obtain SP in the target link. If a significant difference exists, the system will send an alert that indicates the target area is under attack to the network manager. The SDN controller can obtain all the information from the network depending on the OpenFlow protocol. This implementation will focus on the SDN controller.

The SDN controller obtains the topology of the network, such as the link between the switch and the switch, the link between the switch and the node, or the link between the switch and the controller, so we can use this API to determine all the distributions and positions of the links in the network. The SDN controller obtains the flow table from the links. The TSM obtains flows that pass the target area to calculate the link weight of each link, and the SPM obtains input and output flows that pass through the target area.

## 6. Evaluation

This section compares our proposed FDD with other detection methods through experiments. We first introduce the experimental environments, scenarios, performance metrics, and methods to be compared. Then, the effects of detection performance on different parameters, such as the ratio of hybrid attacks, the number of servers in the target area, and the number of switches, are investigated.

For comparison of the detection effect between the FDD and the current detection systems, therefore, the experimental group is the FDD, and the control group is LFADefender (LAD) [21], which detects LFAs, Reinforcing Anti-DDoS Actions in Realtime (RADAR) [24], which detect SEAs, and Combiner (COM), which combines LAD and RADAR. If one of the detection methods detects an attack, COM will identify the situation as an attack.

### 6.1. Experimental Environment

This subsection explains how to set up the experimental environment, including the tools for simulating the network, attacks, and parameters, all experiment scenarios, evaluation of detection performance metrics, and the methods to be compared, are described.

The experiment setup in [21] was used for our testbed, along with Mininet to simulate the DetNet SDN environment and one SDN controller, OpenDayLight. The core of the network is composed of switches in an Ubuntu system 16.04 virtual machine. Because we considered the limitations of the experimental equipment, the bandwidth of all links in our testbed is 10 Mbps. In addition, we set up botnet access requests to the target area in SDN to generate legitimate traffic using iPerf [21] and trafgen [26], which are network tools, to simulate hybrid attacks on the testbed, where we could directly control all hosts. iPerf and trafgen are tools to generate and observe traffic based on the maximum achievable bandwidth on IP networks. This traffic supports different timings, protocols, and buffers, and it can collect corresponding performance results, including throughput, loss, and other parameters. We employed five botnets that played the role of an SEA and LFA and twenty-five hosts as normal usage. The SDN controller and bot were built by a container deployed in several Virtual Private Cloud (VPC) networks in different cloud service providers to emulate the DetNet architecture. The container is a lightweight virtualized service containing only the application and its libraries and dependencies, and VPC is a virtual version of a physical network.

The SEA botnets with trafgen [26] send at least 10,000 SYN packets, and LFA botnets send traceroute packets to the target area to find target links before sending TCP or UDP packets with iPerf [21] at both ends of the target link to congest the target links. During the attack phase, the bandwidth of attack traffic from bots is 2 Mbps. At the same time, the remaining twenty-five normal hosts randomly access the three servers in the target area because attacks will exist simultaneously with normal usage in the real world.

### 6.2. Scenarios

By default, we set up the topology with seven switches in the SDN, three servers in the target area, and five botnets as attackers in the SDN to combine SEAs and LFAs under different ratios to achieve the effect of hybrid attacks. *R* is the hybrid ratio and is defined as *LB/SB*, where *LB* is the number of LFA botnets, and SB is the number of SEA botnets. This research experimentally tested the effectiveness of one to five servers in the target area because the number of servers could be changed. This study randomly generated attacking and normal traffic based on a fuzzy testing methodology to ensure all experiments in this section have sufficient samples/scenarios regarding hybrid attacking and normal traffic.

**Figure 4:**
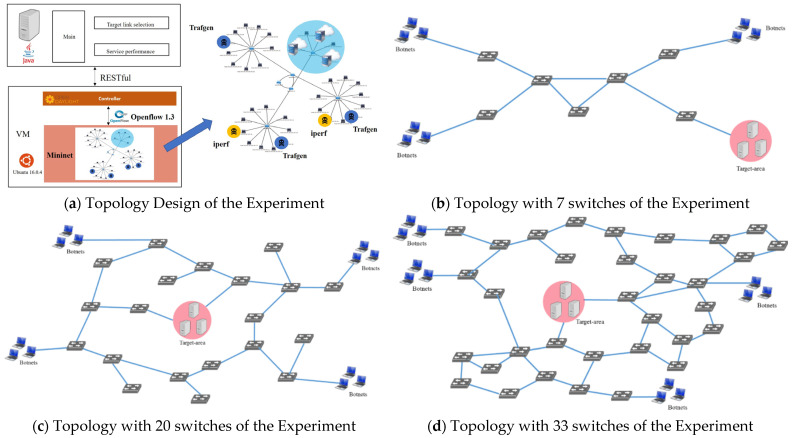
Experimental topology.

Finally, we tested three types of topologies [21] where hybrid attacks occurred to detect an effect. Figure 4a demonstrates the topology design. Figure 4b has seven switches and one SDN controller, which is the core of the network. Figure 4c has 20 switches and 1 SDN controller. Figure 4d has 33 switches and 1 SDN controller.

### 6.3. Performance Metrics

In the evaluation effect part, three measures are considered, namely detection accuracy, false-positive rate (FPR), and false-negative rate (FNR), which are classified by the confusion matrix shown in Table 3. True positive (TP) is the number of attacksbeing attacked; false negative (FN) is the number of attacks seen as normal usagebeing attacked, false positive (FP) is the number attacks under normal usage situations, and true negative (TN) is the number of attacks seen as normal usage under normal usage situations. Detection accuracy means that the detection system can correctly identify whether it is an attack or normal. For example, according to Formula (4), FPR means that the detection system wrongly identifies attacks under normal usage situations according to Formula (5). FNR means that the detection system cannot identify the attacks and miss rate according to Formula (6).

(4) $$Accuracy=\frac{TP+TN}{TP+TN+FP+FN}\;\;\;\;\;\;$$

(5) $$FPR=\frac{FP}{FP+TN}$$

(6) $$FNR=\frac{FN}{FN+TP}\;\;$$

### 6.4. Effects of Hybrid Ratio

**Figure 5:**
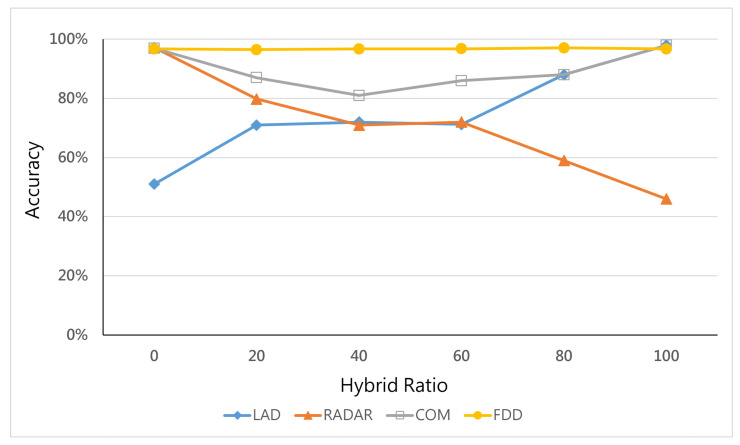
Accuracy vs. hybrid ratio.

Figure 5 demonstrates the performance of different ratios of hybrid attacks in a topology with seven switches and three servers. When the ratio of hybrid attacks increases, the detection accuracy of LAD, RADAR, and COM becomes inferior. FAD’s average detection accuracy is 75%, RADAR’s average detection accuracy is 72.22%, and COM’s average detection accuracy is 90%. The detection accuracy of the FDD is the most stable, with an average detection accuracy of 96.67%, which is better than previous studies.

**Figure 6:**
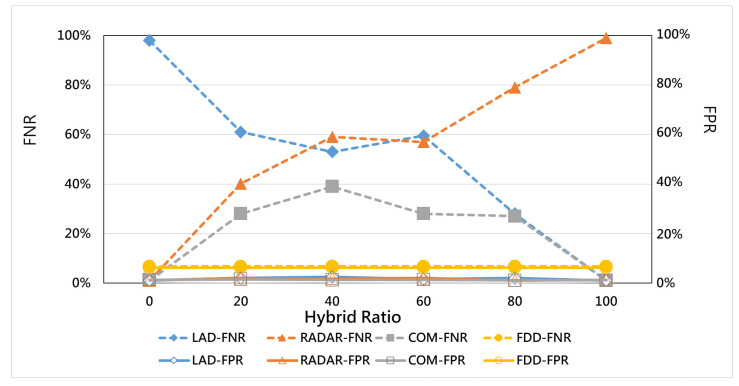
FNR and FPR vs. hybrid ratio.

We further explored the type I and type II errors of all studies by observing the false-positive rate (FPR) and false-negative rate (FNR). A type I error is the false rejection of the null hypothesis (FPR) such as “normal traffic is detected as a hybrid attack”, while a type II error is the false acceptance of the null hypothesis (FNR) such as “a hybrid attack is detected as normal traffic”. Figure 6 describes the reason causing the difference in average detection accuracy. FDD-FPR and FDD-FNR are both lower than 10%, which is better than all the other detection systems because the FDD detects target link congestion in small-size topologies more easily.

It should be noted that the accuracy rate of previous studies shown in Figure 5, which are LAD, RADAR, and COM, dropped between the 20% and 80% hybrid ratio. On the other hand, the FPR and FNR of previous studies, which are LAD, RADAR, and COM, changed abnormally between the 20% and 80% hybrid ratio. This is because the hybrid attacks were a combination of an SEA and LFA. Previous detection systems only depend on one value that increases under a single-type attack, and they cannot identify hybrid attacks. However, our proposed FDD maintains high accuracy and a low FPR/FNR because it identifies the attacks according to flows through the target area, which indicates the differentiation of the service input and output flows. As long as the service is affected by the attack, whether it is link congestion or server collapse, the FDD will be determined as an attack. We can infer that the FDD can solve the problem that single-type detection systems cause a miss rate when hybrid attacks occur.

### 6.5. Effects of the Number of Servers in Target Area

This experiment attempts to explore the performance of all methods detecting the hybrid attack and the synergy effect of different numbers of servers in the target area. The environment setting is that the hybrid ratio is equal to 0.4 in the topology with seven switches.

**Figure 7:**
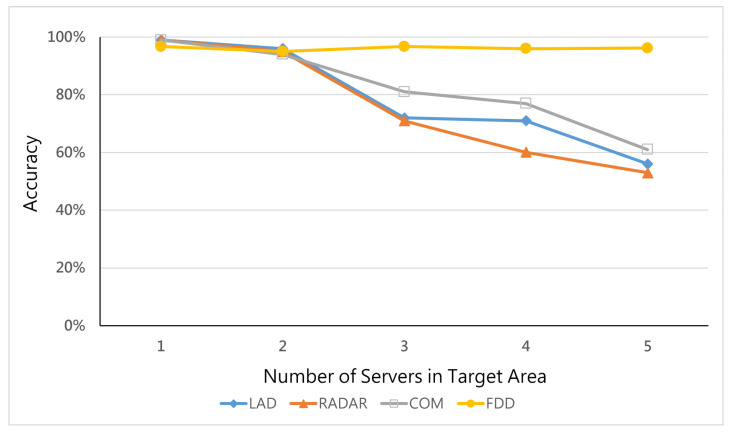
Accuracy vs. number of servers in the target area.

**Figure 8:**
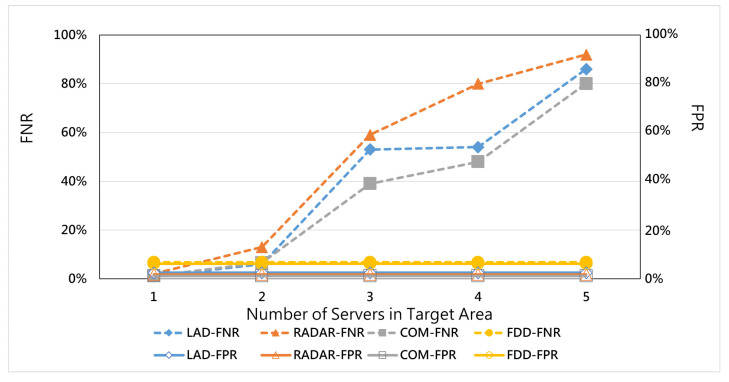
FNR and FPR vs. number of servers in the target area.

Figure 7 shows that, when there is only one server in a target area, all methods have good detection accuracy above 90% because all these methods merely watch over the traffic related to this server. However, as the number of servers in the target area increases, the traditional detection methods, i.e., LAD, RADAR, and COM, experience low detection accuracy. On the other hand, our proposed FDD still has a stable detection accuracy performance. Figure 8 also supports the above argument that traditional studies experience higher type 2 errors (FNR), as when a hybrid attack is detected as normal traffic, the number of servers rises. We can infer that traditional methods use a single observation point to detect attacks without a self-adjusting ability when the number of servers is changed. The FDD is based on multiple target links to detect hybrid attacks, so it is hardly affected.

### 6.6. Effects of the Number of Switches

**Figure 9:**
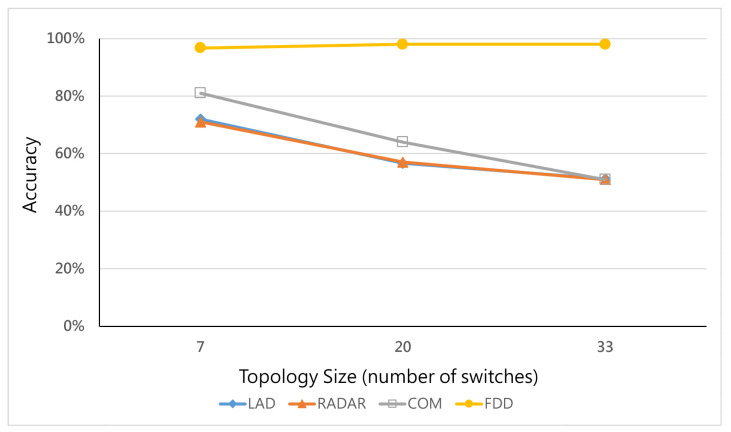
Accuracy vs. number of switches.

**Figure 10:**
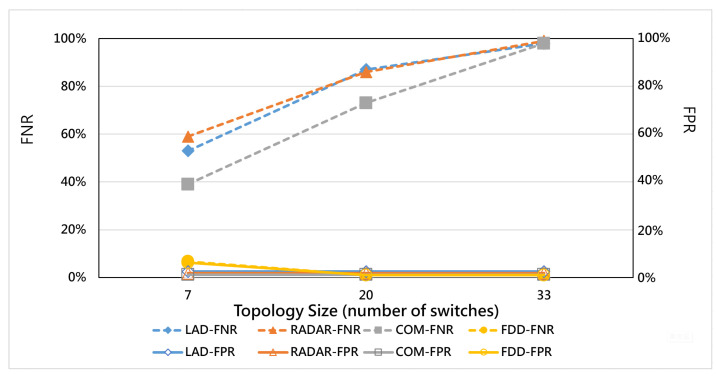
FNR and FPR vs. number of switches.

This section further explores the performance of all methods when facing different scales of topology. In this experiment, the hybrid ratio is equal to 0.4, and the three servers in the target area are under different numbers of switches. The results are shown in Figure 9. The FDD has a 98.9% average detection accuracy rate for detecting hybrid attacks under each topology. However, the detection accuracy of traditional methods, i.e., LAD, RADAR, and COM, becomes worse when the topology becomes larger. This may be due to the inability of traditional studies to detect hybrid attacks, as shown in Figure 10, leading to higher type 2 errors (FNR) when the topology becomes larger and the synergy effect becomes deteriorated.

## 7. Conclusions and Future Work

In this study, we proposed the FDD, an approach against Hybrid DDoS attacks that leverages SDN, to effectively detect Hybrid DDoS attacks according to the experimental results. In the detection system, we deployed the FDD on OpenDayLight and evaluated it on Mininet, which simulates a real network environment.

The FDD first applies a fuzzy-based mechanism, Target Link Selection, to determine the most valuable links for the DDoS link/server attacker, and then it statistically evaluates the traffic pattern flowing through these links. Furthermore, the contribution of this study is to deploy the FDD in the SDN controller OpenDayLight to implement a Hybrid DDoS attack detection system. The experimental results show that the FDD has superior detection accuracy (above 90%) than traditional methods under the situations of different ratios of Hybrid DDoS attacks and different types and scales of topology.

In future work, we plan to extend our work to Artificial Intelligence (AI) technology and explore the best setting of the moderator *k* in our proposed FDD. Furthermore, we will explore the possibility of developing an AI robustness test methodology to verify that the artificial intelligence model has been fully trained to ensure robustness and effectiveness.

## Figures and Tables

**Table 1:** DDoS attack detection comparison table.

Method	Attack Type	Flow/Packet-Based
Calculate SYN response rate [8]	SYN	Packet-based
Use flow to calculate IP repetition rate [9]	DNS	Flow-based
Calculate entropy to detect [10]	SYN	Flow-based
Use the dynamic resource allocation mechanism [11]	SYN	Flow-based
Use ML to train the packet feature [12]	DNS	Packet-based
Use ML to train the packet feature [13]	DNS	Packet-based
Analyze server logs to train an abnormal packet [14]	HTTP	Packet-based
Use correlation IP and traffic to detect [15]	HTTP	Flow-based
Analyze user trace logs to train model [16]	HTTP	Packet-based
Probe to measure link utilization [19]	Crossfire	Flow-based
Analyze node-to-node traffic variation topology [20]	Crossfire	Packet-based
Use flow to measure link utilization [21]	Crossfire	Flow-based
Analyze traceroute packet and reroute [22]	Crossfire	Packet-based
Analyze traceroute packet and reroute [23]	Crossfire	Packet-based
Use correlation to cluster traffic to measure link utilization [24]	Hybrid	Flow-based

**Table 2:** Used notations.

Notation	Description
*T*	Number of switches
*SW =* $\left\{sw_{j}^{}|\;1\;\leq j\leq T\right\}$	*SW*: switches in SDN; $sw_{j}^{}$: which switch
*TA* = $\left\{ta_{i}^{}|\;i\;\geq \;1\right\}$	*TA*: servers in the target area; $ta_{i}^{}$: which server
*F* = { $f_{m}^{}|\;m\;\geq \;1$}	*F*: flows in SDN; $f_{m}^{}$: which flow
*α*	Ratio of the link as a target link
*L =* $\left\{sw_{j,k}^{}|\;1\;\leq \;k\leq T\;\right\}$	*L*: links in SDN; $sw_{j,k}^{}$: which link
*TL*	Target links in SDN
*SP*	Service performance

**Table 3:** Confusion matrix.

	Yes (Fact)	No (Fact)
Yes (Prediction)	TP	FP
No (Prediction)	FN	TN
